# The role of psychosocial factors in explaining sex differences in major depression and generalized anxiety during the COVID-19 pandemic

**DOI:** 10.1186/s12889-022-13954-8

**Published:** 2022-08-17

**Authors:** Frédérique Vallières, Jamie Murphy, Orla McBride, Mark Shevlin, Brynne Gilmore, Áine Travers, Ann Nolan, Sarah Butter, Thanos Karatzias, Richard Bentall, Philip Hyland

**Affiliations:** 1 Trinity Centre for Global Health, University of Dublin, Trinity College, Dublin, Ireland; 2 School of Psychology, Ulster University, Coleraine, Northern Ireland; 3 Education and Innovation in Health Systems, School of Nursing, Midwifery and Health Systems, UCD Centre for Interdisciplinary Research, University College Dublin, Dublin, Ireland; 4 Department of Psychology, University of Sheffield, Cathedral Court, 1 Vicar Lane, Sheffield, S1 2LT UK; 5 School of Health and Social Care, Edinburgh Napier University, Edinburgh, Scotland; 6 Department of Psychology, Maynooth University, Kildare, Ireland

**Keywords:** Sex-differences, Depression, Anxiety, COVID-19 pandemic

## Abstract

**Background:**

Understanding how pandemics differentially impact on the socio-protective and psychological outcomes of males and females is important to develop more equitable public health policies. We assessed whether males and females differed on measures of major depression and generalized anxiety during the COVID-19 the pandemic, and if so, which sociodemographic, pandemic, and psychological variables may affect sex differences in depression and anxiety.

**Methods:**

Participants were a nationally representative sample of Irish adults (*N* = 1,032) assessed between April 30^th^ to May 19^th^, 2020, during Ireland’s first COVID-19 nationwide quarantine. Participants completed self-report measures of anxiety (GAD-7) and depression (PHQ-9), as well as 23 sociodemographic pandemic-related, and psychological variables. Sex differences on measures of depression and anxiety were assessed using binary logistic regression analysis and differences in sociodemographic, pandemic, and psychological variables assessed using chi-square tests of independence and independent samples t-tests.

**Results:**

Females were significantly more likely than males to screen positive for major depressive disorder (30.6% vs. 20.7%; *χ*^*2*^ (1) = 13.26, *p* < .001, OR = 1.69 [95% CI = 1.27, 2.25]), and generalised anxiety disorder (23.3% vs. 14.4%; *χ*^*2*^ (1) = 13.42, *p* < .001, OR = 1.81 [95% CI = 1.31, 2.49]). When adjusted for all other sex-varying covariates however, sex was no longer significantly associated with screening positive for depression (AOR = 0.80, 95% CI = 0.51, 1.25) or GAD (AOR = 0.97, 95% CI = 0.60, 1.57).

**Conclusion:**

Observed sex-differences in depression and anxiety during the COVID-19 pandemic in the Republic of Ireland are best explained by psychosocial factors of COVID-19 related anxiety, trait neuroticism, lower sleep quality, higher levels of loneliness, greater somatic problems, and, in the case of depression, increases in childcaring responsibilities and lower trait consciousnesses. Implications of these findings for public health policy and interventions are discussed.

## Background

Whether repeatedly observed sex differences in Major Depressive Disorder (MDD) and General Anxiety Disorder (GAD) are primarily attributable to biological differences between the sexes or are better explained by socio-cultural factors is of considerable debate within the literature [1]. For example, while greater sex differences are found in nations with greater gender equity [2], inter-cohort narrowing on levels of major depression and substance misuse have also been noted in the context of changes to the traditionality of female gender roles [3]. The sudden and rapid socio-environmental changes enacted during the COVID-19 pandemic offer a unique opportunity to further explore whether, and if so, which sociodemographic, pandemic, and psychological variables are associated with sex differences in depression and anxiety.

Consistent with global trends suggesting that women are at increased risk of MDD and GAD [1], women remain disproportionally mentally and emotionally affected during the COVID-19 pandemic [4–8]. An analysis of the United Kingdom (UK) Household Longitudinal Survey, for example, found the rate of decline in general mental health for women during the COVID-19 pandemic to be twice that experienced by men [9].

Sex-differences have also been observed across a number of other pandemic-related variables. Globally, females comprise the majority (70%) workers in the health and social sector [10, 11]. Females are also more likely to be unemployed during the pandemic [12], or work fewer hours or be employed in low-paid and precarious employment, increasing their vulnerability to job loss [11]. Females also continue to do more of the household and childcare work, taking on more care-role responsibilities outside and within a pandemic [13, 14]. For working women, such additional responsibilities represent important opportunity costs, at the expense of work productivity and potential career-advancement opportunities [15]. Reports of increased intimate partner violence, of which the majority of victims are female [16], have also been noted during the lockdown period [17, 18]. Males, on the other hand, are more likely to be hospitalised, admitted to intensive care, and succumb to death from COVID-19 [19].

Despite noted differences, gender analyses within responses to the current and previous pandemics are noticeably absent from related public health documents, debates, policies, and data tracking processes [19, 20]. Understanding *how* pandemics differentially impact on the socio-protective and psychological outcomes of males and females is important to develop more targeted and equitable public health policies [21]. Accordingly, the current study had three objectives. First, we sought to determine if the proportion of males and females who screened positive for MDD and GAD significantly differed during Ireland’s first nationwide lockdown. In line with the extant literature, we hypothesised that significantly more women than men would screen positive for MDD and GAD. Second, we sought to identify whether males and females significantly differed across a range of sociodemographic, psychological, and pandemic-related variables. Here again, we hypothesised that males and females would differ significantly on a number of these variables. Third, we assessed if hypothesised sex differences in MDD and GAD would be affected – attenuated or exaggerated – by controlling for other sex-varying sociodemographic, psychological, and pandemic-related variables. While we did not formulate a specific hypothesis relating to this objective, if MDD and GAD during the pandemic are predominately attributable to differences on these variables, then we expect any observed sex differences to partly, or wholly, reduce once these sex-differing variables are controlled for.

## Methods

### Participants and procedure

This study was conducted as part of the Irish arm of the COVID-19 Psychological Research Consortium (C19PRC) Study—an ongoing, longitudinal project assessing the psychosocial impact of the COVID-19 pandemic across multiple nations [22]. To date, five waves of data have been collected. Data from Wave 2 of the C19PRC Study, collected April 30^th^ to May 19^th^, 2020 during Ireland’s first nationwide lockdown, was used in this study.

The survey company Qualtrics was employed to recruit participants from traditional, actively managed, double-opt-in research panels via email, SMS, or in-app notifications. Participants were invited to follow a link where they were provided with a description of the study and, if willing to participate, asked to provide informed consent. Participants (*N* = 1,032) had to be aged 18 years or older, resident in the Republic of Ireland, could read and write in English, and were selected using quota sampling methods to generate a sample representative of the adult general population of Ireland in terms of sex, age, and geographical distributions as per the most recent (2016) census [23]. All measures were completed online, with a median completion time of 23.58 min. Participants received financial reimbursement from Qualtrics for their time. Sociodemographic characteristics of the sample are presented in Table 1.

**Table 1:** Sociodemographic and pandemic-related characteristics of the sample (*N* = 1,032)

	%	Mean	SD
*Sex*			
Female	51.9		
Male	47.8		
‘Other’	0.3		
Age		44.86	15.74
Irish Nationality	71.6		
Grew up in Ireland	79.1		
*Living location*			
City	20.3		
Suburb	21.4		
Town	28.5		
Rural	29.8		
Irish Ethnicity	75.0		
In a committed relationship	70.7		
Number of children in the household		1.70	1.02
Number of adults in the household including oneself		2.44	1.09
Living alone	12.8		
Attended university or third-level education	71.0		
*Employment status*			
Full-time (self)/employed	42.9		
Part-time (self)/employed	18.2		
Retired	16.6		
Unemployed	22.4		
*2019 income*			
€0-€19,999	22.0		
€20,000-€29,999	20.2		
€30,000-€39,999	19.9		
€40,000-€49,999	13.0		
€50,000 +	25.0		
*Diagnosis of a chronic illness*			
Self	24.1		
Family member	34.0		
*COVID-19 status*			
Suspected or confirmed infection—self	1.2		
Suspected or confirmed infection—loved one	3.5		
Someone close to you died of COVID-19	4.2		
*Changes to homelife due to COVID-19 pandemic*			
Increased child caring responsibilities	19.7		
Increased housework responsibilities	32.9		
Increased care of elderly relatives	16.9		
Increased feeling of being unsafe in the home	7.9		
Inceased occurrence of intimate partner violence	4.1		
Anxiety related to the COVID-19 pandemic (0 to 100 scale)		61.10	26.60
Perceived risk of COVID-19 infection (0 to 100 scale)		37.62	24.42
Finacial worries due to COVID-19 (1 to 10 scale)		5.37	2.92
Income change due to COVID-19 (-100% to + 100% scale)		-9.74	28.61

*SD* Standard deviation

### Measures

#### Depression and anxiety

##### MDD

The Patient Health Questionnaire-9 [24] (PHQ-9) measures the nine symptoms of MDD, as described in the DSM-5 [25]. Participants indicated how often they have been bothered by each symptom over the last two weeks using a four-point Likert scale that ranges from ‘Not at all’ (0) to ‘Nearly every day’ (3). Scores ≥ 10 have adequate sensitivity (0.85) and specificity (0.89) in identifying those who meet diagnostic criteria. The psychometric properties of the PHQ-9 scores are widely supported [26], and the internal reliability in the current sample was excellent (α = 0.91).

##### GAD

The Generalized Anxiety Disorder 7-item Scale [27] (GAD-7) asks participants to indicate how often they have been bothered by each symptom over the last two weeks using a four-point Likert scale (0 = ‘Not at all’, to 3 = ‘Nearly every day’). Scores ≥ 10 have adequate sensitivity (0.89) and specificity (0.82) in identifying persons who meet diagnostic criteria for GAD. The GAD-7 has been shown to produce reliable and valid scores in community studies [28], and the internal reliability in the current sample was excellent (*α* = 0.94).

#### Sociodemographic and COVID-19 pandemic-related variables

Twenty-three sociodemographic and COVID-19 pandemic-related variables were used in this study (see Table 1).

#### Psychological variables

##### Personality traits

The Big-Five Inventory [29] (BFI) measures the traits of openness, conscientiousness, extraversion, agreeableness, and neuroticism. Each trait was measured by two items using a five-point Likert scale that ranges from ‘strongly disagree’ (1) to ‘strongly agree’ (5). Higher scores reflect higher levels of each personality trait, and the BFI has produces scale scores with good reliability and validity [29]. Internal reliability estimates are not reported given coefficient alpha is inappropriate for demonstrating internal consistency where only two items are used [30].

##### Internal locus of control

The three-item ‘Internal’ subscale of the Locus of Control Scale [31] was used to assess the extent to which people believe that they have control over the things that occur in their life (e.g., ‘My life is determined by my own actions’). The three questions use a seven-point Likert scale ranging from ‘strongly disagree’ [1] to ‘strongly agree’ [7]. Higher scores reflect higher levels of internal locus of control. The internal reliability of the scale scores in this sample was acceptable (*α* = 0.77).

##### Identification with others

The Identification with all Humanity Scale [32] (IWAHS) is a nine-item scale where people respond to three statements with reference to three groups: people in my community, people from Ireland, and all humans everywhere. The response scale ranged from ‘not at all’ [1] to ‘very much’ [5], where higher scores reflect greater identification with others. The internal reliability of the scale scores in this sample was excellent (*α* = 0.93).

##### Religious beliefs

Respondents indicated their agreement to eight statements from the Monotheist and Atheist Beliefs Scale [33]. Response options ranged from ‘strongly disagree’ (1) to ‘strongly agree’ (5). Atheism oriented statements (e.g., ‘Moral judgments should be based on respect for humanity rather than religious doctrine’) were reverse scored and summed with monotheist items to produce a total score, where higher scores reflect religious belief orientation. The psychometric properties of the scale have been previously supported [33], and the internal reliability in the current sample was good (α = 0.81).

##### Intolerance of uncertainty

The Intolerance of Uncertainty scale [34] (US) includes 12 items answered using a five-point Likert scale ranging from ‘not at all characteristics of me’ [1] to ‘entirely characteristic of me’ [5]. Higher scores reflect increased levels of intolerance of uncertainty. The psychometric properties of the IUS scale is widely supported [34]. The internal reliability of the IUS scores in the current sample was good (*α* = 0.88).

##### Loneliness

The three-item Loneliness Scale [35] was designed for use in large-scale population surveys. Respondents are asked to indicate how often they feel that they lack companionship; left out; and isolated from others. Responses are scored using a three-point scale including ‘hardly ever’ (1), ‘sometimes’ (2), and ‘often’ (3). Higher scores reflect higher levels of loneliness. The internal reliability of the scale scores in this sample was good (*α* = 0.87).

##### Somatic problems

The Patient Health Questionnaire-15 [36] (PHQ-15) a self-report measure that asks participants how often they have been bothered by a list of 15 commonly reported physical complaints over the last two weeks. We excluded the ‘menstrual problems’ item due to its sex-specific nature that would preclude analysis of the entire sample. The response options are ‘Not bothered at all’ (0), ‘Bothered a little’ (1), and ‘Bothered a lot’ (2). A total scale score of the 14 items was computed with higher scores reflecting greater somatic problems. The internal reliability of the scale in this sample was good (*α* = 0.83).

##### Sleep Quality

The Sleep Condition Indicator [37] (SCI) is an eight-item measure of different types of sleep problems including sleep continuity, sleep satisfaction, severity of sleep problems, and daytime functioning. Items are scored on a four-point Likert scale with scores ranging from 0–32. Higher scores reflect better sleep quality. The SCI scale has been shown to produce reliable and valid scores [37], and the internal reliability in this sample was good (*α* = 0.88).

#### Data analysis

The analytic strategy included three linked phases. First, differences between the proportion of males and females who screened positive for MDD and GAD, respectively, were assessed using binary logistic regression analysis. Odds ratios (OR) with 95% confidence intervals were calculated to quantify the magnitude of these differences. Second, differences between males and females on all sociodemographic, pandemic, and psychological variables were assessed using chi-square tests of independence (for categorical variables) and independent samples t-tests (for continuous variables). Phi coefficients and Cohen’s d values were calculated to quantify the magnitude of differences for the categorical and continuous variables, and interpreted according to their respected conventions [38]. Finally, sex and all sex-varying variables were added to a binary logistic regression model as predictors of screening positive for MDD and GAD, respectively. Adjusted odds ratios (AOR) with 95% confidence intervals were calculated. There was minimal missing data (0.3%), and this was managed using listwise deletion.

#### Ethical considerations

All research was performed in accordance with the Declaration of Helsinki. Ethical approval was granted by the University of Sheffield (No. 033759) and Ulster University.

## Results

### Sex differences in MDD and GAD

In the full sample, 25.9% (95% CI = 23.2, 28.5%) screened positive for MDD, and 19.0% (95% CI = 16.6, 21.4) screened positive for GAD. Women were significantly more likely than men to screen positive for MDD (30.6% vs. 20.7%; *χ*^*2*^ (1) = 13.26, *p* < 0.001, OR = 1.69 [95% CI = 1.27, 2.25]), and GAD (23.3% vs. 14.4%; *χ*^*2*^ (1) = 13.42, *p* < 0.001, OR = 1.81 [95% CI = 1.31, 2.49]).

### Sex differences on sociodemographic, pandemic, and psychological variables

Males and females significantly differed on 17 sociodemographic, pandemic, and psychological variables (see Table 2). Women were significantly more likely than men to live in a rural location and less likely to live in a town; less likely to be in a committed relationship; less likely to be employed full-time or be retired, and more likely to be employed part-time; more likely to be earning less than €20,000 a year and €30,000-€39,999 a year, and less likely to be earning €50,000 or more a year; more likely to have a family member with a chronic illness; and more likely to have experienced an increased level of childcare and housework responsibilities due to the COVID-19 pandemic.

**Table 2:** Sex differences on all sociodemographic, pandemic, and psychological variables

	Males	Females	Main effect
**Categorical variables**	%	%	χ^2^ (df), p, φ
Irish nationality	73.8	70.0	1.90 (1), .168, .04
Grew up in Ireland	79.7	78.7	0.15 (1), .697, .01
Irish ethnicity	76.9	73.7	1.40 (1), .238, .04
Living location			**10.39 (3), .015, .10**
City	21.5	18.8	
Suburb	21.5	21.5	
Town	31.6	25.7^a^	
Rural	25.4	34.0^a^	
In a relationship	76.1	66.0	**12.48 (1), < .001, .11**
Living alone	13.6	11.9	0.65 (1), .420, .03
Attended university	72.2	69.8	0.74 (1), .390, .03
Employment status			**62.20 (3), < .001, .25**
Full-time employed	52.5	34.1^a^	
Part-time employed	11.4	24.1^a^	
Unemployed	16.6	27.8^a^	
Retired	19.5	14.0^a^	
2019 income level			**54.65 (4), < .001, .23**
€0-€20,000	16.8	26.9^a^	
€20,000-€29,999	18.5	21.6	
€30,000-€39,999	15.8	23.7^a^	
€40,000-€49,999	15.0	11.2	
€50,000 or above	33.9	16.6^a^	
Chronic illness—self	26.0	22.6	1.61 (1), .205, .04
Chronic illness—family	29.2	38.6	**10.12 (1), .001, .10**
COVID-19 infection—self	1.3	1.2	0.05 (1), .829, .01
COVID-19 infection—family	3.2	3.5	0.07 (1), .791, .01
COVID-19 death	5.3	3.2	2.83 (1), .092, .05
Increased childcare	17.0	22.0	**4.03 (1), .045, .06**
Increased housework	28.4	36.9	**8.50 (1), .004, .09**
Increased elderly care	16.6	17.2	0.05 (1), .820, .01
Increased unsafe	6.3	9.5	3.65 (1), .056, .06
Increased IPV	4.9	3.4	1.45 (1), .221, .04
**Continuous variables**	Mean (SD)	Mean (SD)	t, p, Cohen’s d
Age	48.30 (14.95)	41.73 (15.85)	**6.82, < .001, .43**
Number of children in home	1.73 (1.07)	1.67 (0.97)	0.95, .344, .06
Number of adults in home	2.28 (1.01)	2.44 (1.19)	**2.35, .019, .14**
COVID-19 anxiety	58.60 (26.87)	63.37 (26.21)	**2.88, .004, .18**
Perceived risk of infection	37.06 (23.89)	38.05 (24.94)	0.65, .515, .04
Financial worries	5.29 (2.85)	5.43 (3.00)	0.77, .441, .05
Income change	-8.77 (27.92)	-10.61 (29.22)	1.03, .303, .06
Openness	6.67 (1.65)	6.64 (1.66)	0.32, .747, .02
Conscientiousness	8.06 (1.71)	8.39 (1.76)	**2.98, .003, .19**
Extraversion	6.07 (1.88)	6.20 (1.93)	1.03, .302, .07
Agreeableness	7.00 (1.61)	7.03 (1.66)	0.25, .799, .02
Neuroticism	5.29 (2.00)	6.02 (2.07)	**5.77, < .001, .36**
Internal locus of control	11.83 (4.56)	12.13 (4.25)	1.10, .270, .07
Identification with others	31.77 (7.60)	33.32 (7.13)	**3.38, .001, .21**
Religious beliefs	22.32 (6.60)	23.27 (5.85)	**2.44, .015, .15**
Intolerance of uncertainty	38.29 (12.28)	37.46 (12.65)	1.07, .287, .07
Loneliness	4.84 (1.84)	5.31 (1.90)	**4.01, < .001, .25**
Somatic problems	4.68 (4.42)	6.22 (4.62)	**5.45, < .001, .34**
Sleep quality	22.71 (7.67)	19.55 (8.09)	**6.43, < .001, .40**

^a^indicates group differences; *χ*^*2*^ Chi-square test of independence, *df* Degrees of freedom, *p* Statistical significance, *φ* Phi coefficient, *t* Independent samples t-test; all t-tests have 1027 degrees of freedom; statistically significant main effects are in bold

Regarding the continuous variables, women were significantly younger than men, and lived in a home with more adults. Women also had significantly higher levels of COVID-19 related anxiety, trait conscientiousness, trait neuroticism, identification with others, religious beliefs, feelings of loneliness, and somatic complaints. Additionally, women also had significantly lower levels of sleep quality.

### Adjusted predictors of MDD and GAD

The binary logistic regression model predicting screening positive for MDD was statistically significant (*χ*^*2*^ (25) = 488.62, *p* < 0.001) and correctly classified 85.6% of people. When adjusted for all other sex-varying covariates, sex was no longer significantly associated with screening positive for MDD (AOR = 0.80, 95% CI = 0.51, 1.25). Nine variables remained significantly associated with screening positive for MDD including younger age, living in a city versus a town, increased childcare responsibilities due to the pandemic, higher levels of anxiety about the pandemic, lower levels of trait conscientiousness, higher levels of trait neuroticism, higher levels of loneliness, higher levels of somatic problems, and lower levels of sleep quality (see Table 3).

**Table 3:** Binary logistic regression results predicting screening positive for Major Depressive Disorder (MDD) and Generalized Anxiety Disorder (GAD)

	**Major depression**			**Generalized anxiety**		
	AOR	95% CI		AOR	95% CI	
Sex (Female)	0.799	0.513	1.245	0.970	0.601	1.565
Age	**0.963**	**0.944**	**0.981**	**0.968**	**0.949**	**0.988**
Living location (City)						
Suburb	0.922	0.509	1.670	0.552	0.296	1.032
Town	**0.533**	**0.295**	**0.961**	**0.400**	**0.215**	**0.746**
Rural	0.880	0.489	1.584	0.629	0.344	1.151
Not in a relationship	0.888	0.527	1.497	1.008	0.583	1.743
Employment status (Full-time employed)						
Part-time employed	0.857	0.472	1.557	0.745	0.400	1.387
Unemployed	1.304	0.761	2.235	0.719	0.404	1.281
Retired	0.818	0.337	1.984	0.674	0.248	1.832
2019 income level (€0-€20,000)						
€20,000-€29,999	1.175	0.629	2.193	1.023	0.531	1.969
30,000-€39,999	0.692	0.358	1.338	0.514	0.252	1.047
€40,000-€49,999	0.887	0.403	1.952	0.748	0.326	1.716
€50,000 or above	1.440	0.738	2.811	1.082	0.531	2.207
Number of adults in the home	0.889	0.737	1.073	0.857	0.702	1.045
Chronic illness of family member	0.885	0.577	1.357	0.659	0.415	1.046
Increased childcare	**1.894**	**1.123**	**3.194**	1.600	0.924	2.768
Increased housework	0.905	0.557	1.470	1.531	0.917	2.556
COVID-19 anxiety	**1.018**	**1.009**	**1.027**	**1.026**	**1.016**	**1.037**
Conscientiousness	**0.850**	**0.747**	**0.967**	0.976	0.854	1.114
Neuroticism	**1.132**	**1.014**	**1.263**	**1.405**	**1.244**	**1.587**
Identification with others	0.979	0.950	1.008	1.005	0.974	1.037
Religious beliefs	1.012	0.977	1.048	0.985	0.948	1.023
Loneliness	**1.424**	**1.251**	**1.621**	**1.401**	**1.224**	**1.604**
Somatic problems	**1.158**	**1.102**	**1.218**	**1.145**	**1.089**	**1.204**
Sleep quality	**0.882**	**0.855**	**0.909**	**0.927**	**0.899**	**0.957**

*AOR* Adjusted odds ratio, *95% CIs* 95% Confidence intervals; statistically significant associations (*p* < .05) are in bold

The binary logistic regression model predicting screening positive for GAD was also statistically significant (*χ*^*2*^ (25) = 387.10, *p* < 0.001) and correctly classified 87.8% of people. The effect for sex was no longer statistically significant when adjusted for the sex-varying covariates (AOR = 0.97, 95% CI = 0.60, 1.57). Seven variables were significantly associated with screening positive for GAD including younger age, living in a city versus a town, higher levels of anxiety about the pandemic, higher levels of trait neuroticism, higher levels of loneliness, higher levels of somatic problems, and lower levels of sleep quality (see Table 3).

## Discussion

Consistent with large-scale population assessments of the prevalence of depression and anxiety globally [39–41], we found that females in Ireland were more likely to screen positive for MDD and GAD during the COVID-19 pandemic. Moreover, female respondents were more likely to be younger in age, unemployed or in part-time employment, and to be in the lowest income bracket. Female respondents also scored higher on the personality trait of neuroticism, and endorsed greater feelings of loneliness, more somatic problems, and poorer sleep quality during the COVID-19 pandemic. Once all variables where males and females were found to differ were accounted for however, sex was no longer associated with MDD and GAD status. Instead, both of these disorders were associated with age (i.e. being younger), living location (i.e. living in a town was associated with lower risk of both disorders compared to living in a city), scoring higher on neuroticism, and having higher levels of COVID-19 related anxiety, lower sleep quality, higher levels of loneliness, and greater somatic problems. Additionally, scoring lower on conscientiousness and increased caring responsibilities during the pandemic were associated with screening positive for MDD.

Our findings are largely consistent with the results of Etheridge and Spantig’s longitudinal survey conducted in the UK, who also found that the gender gap in mental health during the pandemic was best explained by increased feelings of loneliness since the onset of the pandemic [9]. Specifically, those who reported a larger number of close friends prior to the pandemic, and greater levels of loneliness during the pandemic, reported greater declines in mental wellbeing [9]. Given that positive social relationships are noted as an important resilience-related factor to mitigate psychological distress during difficult periods,[42] the enactment of physical distancing measures should therefore be met with complementary public health messaging and additional resources to counter the effects of loneliness, as a known risk factor for the development of sleep disturbances [43, 44], increased stress levels [45], as well as a myriad of adverse health—including mental health – effects, and premature mortality [46, 47]. Given the obvious limitations of increasing opportunities for social interaction within the context of a nationwide lockdown, resources should be put towards publicly available interventions that focus on attenuating maladaptive thoughts and social cognitions underlying loneliness [48]. Increasing the availability of mobile or online-based low-intensity cognitive behavioural therapy interventions that leverage already existing and widely-used platforms offer one such promising solution [49].

The bidirectional effects of poor sleep quality—including insomnia, hypersomnia, circadian rhythm disturbance – anxiety, depression, and somatic disturbances are also well noted in the literature [50, 51], whereby even short-term sleep loss and deprivation have been found to lead to temporary changes in mood and cognition and somatic complaints [52]. Changes to daily routines and sleeping habits associated with the pandemic, including difficulties in keeping a consistent sleep schedule, marked decreases in number of hours spent sleeping, difficulties falling or staying asleep, and the development of other sleep disturbances were also noted during nationwide lockdowns in Italy and the UK [53, 54]. It is therefore important to consider how sudden changes to people’s quotidian, including changes in social, sleep and lifestyle behaviours, increased internet use, may influence mental health and stress responses [55]. Health messaging promoting the importance of, in as much as possible, maintaining one’s pre-pandemic routine, including regular sleeping hours, exercise, and moderation of caffeine and alcohol are also recommended.

That reported increases in childcaring responsibilities were associated with depression is consistent with the observation that despite already taking on twice as much care and household work compared to men prior to the pandemic [56], women also took on more childcaring responsibilities following Ireland’s nationwide school and childcare facility closures [57]. A Canadian longitudinal observational study of women also found greater increases in depression and anxiety during the COVID-19 pandemic among those who experienced difficulties in obtaining childcare and balancing home schooling and work-related responsibilities [7]. Similarly, greater feelings of psychological distress during the COVID-19 pandemic were among women with elementary school-age and younger children, compared to women without children and all men in a US population-representative sample [58]. Additional childcaring responsibilities have also notably impacted on employment status and educational disruptions for parents [14]. Prior to the pandemic, Ireland already had the third highest weekly hours of unpaid work for both males and females in the European Union (EU), reflecting the relatively low-level of State involvement in support for caring [56]. That women are more likely to cease or reduce paid work to meet unpaid COVID-19-related childcare needs [59] further emphasises the urgency of policy reform to address the gendered allocation of unpaid care among women in Ireland [60].

The current study is not without limitations. Firstly, as a quota sample, participants in this study do not represent a true random sample of the population of Ireland. The current study may therefore be susceptible to a ‘healthy volunteer’ sampling bias, whereby those who are healthier are more likely to have agreed to take part in this study. Second, and as all mental health assessments were based on self-report rather than clinician administered interviews, this may have resulted in over- or under-estimation of disorder prevalence. Third, although a large number of risk factors were assessed, other known risk factors (e.g., lack of social support; substance misuse) were missing from the study/analysis. Finally, given the cross-sectional nature of the study, we cannot offer evidence as to whether observed levels of loneliness, somatic complaints, and sleep disturbances were due to the pandemic itself.

## Conclusions

Pandemics serve as an important reminder that health outcomes are not solely determined by the biological effects of pathogens. Requisite rapid and substantial changes to our social environment, which may present differently for males and females, also act as important determinants of psychological and physical health. During the first nationwide COVID-19 lockdown in the Republic of Ireland, observed sex-differences in anxiety and depression were primarily explained by the psychosocial factors of age, living location, neuroticism, increased feelings of COVID-19-related anxiety, increased loneliness, somatic complaints, sleep disturbances, and, in the case of depression, increased childcare responsibilities.

## Data Availability

Data is available, upon reasonable request, from the first (FV) or last author (PH).

## Abbreviations

AOR: Adjusted Odds Ratio
BFI: Big Five Inventory
C19PRC: COVID-19 Psychological Research Consortium
EU: European Union
GAD: General Anxiety Disorder
IUS: Intolerance of Uncertainty
IWAHS: Identification with all Humanity Scale
MDD: Major Depressive Disorder
OR: Odds Ratio
PHQ: Patient Health Questionnaire
SCI: Sleep Condition Indicator
UK: United Kingdom
